# Host Cell Restriction Factors Blocking Efficient Vector Transduction: Challenges in Lentiviral and Adeno-Associated Vector Based Gene Therapies

**DOI:** 10.3390/cells12050732

**Published:** 2023-02-24

**Authors:** Ana Sofia Coroadinha

**Affiliations:** 1 iBET, Instituto de Biologia Experimental e Tecnológica, Apartado 12, 2781-901 Oeiras, Portugal; avalente@ibet.pt; 2 Instituto de Tecnologia Química e Biológica António Xavier, Universidade Nova de Lisboa, Av. da República, 2780-157 Oeiras, Portugal

**Keywords:** gene therapy, lentiviral vectors, adeno-associated virus vectors, innate-immune response, host restriction factors, transduction

## Abstract

Gene therapy relies on the delivery of genetic material to the patient’s cells in order to provide a therapeutic treatment. Two of the currently most used and efficient delivery systems are the lentiviral (LV) and adeno-associated virus (AAV) vectors. Gene therapy vectors must successfully attach, enter uncoated, and escape host restriction factors (RFs), before reaching the nucleus and effectively deliver the therapeutic genetic instructions to the cell. Some of these RFs are ubiquitously expressed in mammalian cells, while others are cell-specific, and others still are expressed only upon induction by danger signals as type I interferons. Cell restriction factors have evolved to protect the organism against infectious diseases and tissue damage. These restriction factors can be intrinsic, directly acting on the vector, or related with the innate immune response system, acting indirectly through the induction of interferons, but both are intertwined. The innate immunity is the first line of defense against pathogens and, as such cells derived from myeloid progenitors (but not only), are well equipped with RFs to detect pathogen-associated molecular patterns (PAMPs). In addition, some non-professional cells, such as epithelial cells, endothelial cells, and fibroblasts, play major roles in pathogen recognition. Unsurprisingly, foreign DNA and RNA molecules are among the most detected PAMPs. Here, we review and discuss identified RFs that block LV and AAV vector transduction, hindering their therapeutic efficacy.

## 1. Introduction

Scientific knowledge and technological progress in the last few decades have not only accelerated the discovery of the cellular and molecular mechanisms behind human diseases, but also driven the generation of advanced gene and cell therapies. Gene therapy arose as a promising approach to revolutionize medicine, enabling treatments that provide long-term effects for a wide variety of inherited and acquired diseases. The potential of gene therapy is currently consolidated into effective treatments. Indeed, in the last decade, several gene therapy-based products have been approved in Europe and in the United States of America. Strimvelis, used for treating severe combined immunodeficiency due to adenosine deaminase deficiency (*ADA*-SCID), a rare disease; Luxturna, used for treating patients with inherited retinal disease due to mutations in both copies of the retinal pigment epithelium-specific 65 kDa gene (*RPE65)*; and Kymriah used to treat cancer patients who have acute lymphoblastic leukemia (ALL) are three examples of such products in [1].

Gene therapy medicinal products are based on the use and administration of an active substance that contains or consists of a recombinant nucleic acid, ultimately aiming to regulate, repair, replace, add, or delete a genetic sequence to attain a therapeutic, prophylactic, or diagnostic effect [2]. Thus, gene therapies require the transfer of DNA- or RNA-based molecules into the patient’s cells. Naked nucleic acids cannot efficiently enter cells, are often unstable, and are subject to nuclease degradation. Delivery systems are required. Different types of gene therapy vectors, either viral on non-viral, have and are being developed for this purpose. However, gene therapy vectors face several obstacles in executing their function, namely they must escape host cell RFs. The latter are inhibitory host cell factors, belonging to the cellular innate immune system, or to intrinsic antiviral immunity, which interfere with vector trafficking and expression at diverse steps, from cell entry to nucleic acid translation [3].

Host cell RFs have evolved to protect the organism against infectious diseases and tissue damage. The innate immune response is the first line of defense against pathogens and, as such, the cells involved in it are well equipped with RFs that detect PAMPs and damage-associated molecular patterns (DAMPs). These cells include macrophages, neutrophils, eosinophils, basophils, mast cells, and dendritic cells that are derived from myeloid progenitors, but also, natural killer cells derived from lymphoid stem cells. In addition, some nonprofessional cells, such as epithelial cells, endothelial cells, and fibroblasts, also play major roles in pathogen recognition during the innate immune response. The cells of the host recognize PAMPs via germ line-encoded pattern recognition receptors (PRRs) [4]. These cells, encoding PRRs, may be difficult to transduce when the target of therapy, as they are sources of danger signals to other cells, upregulating the expression of their non-constitutive RFs. Thus, in in vivo therapies, the off-targeting of cells from the innate immune system, may indirectly hamper transduction of the targeted cells.

**Figure 1:**
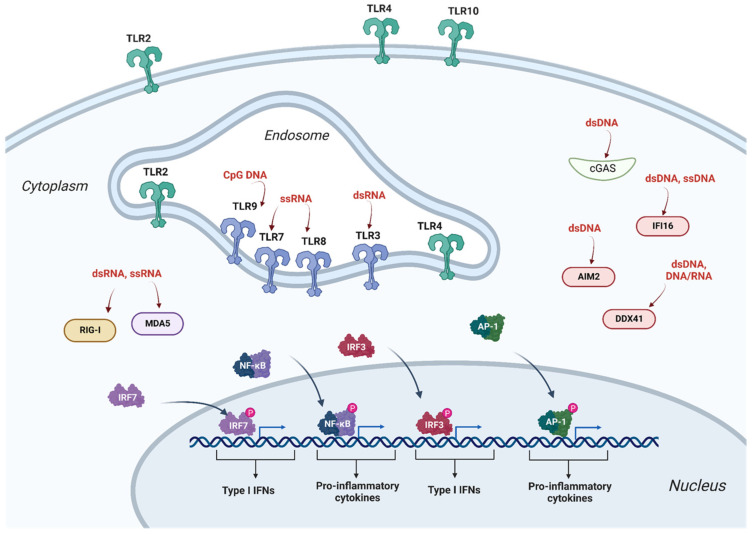
Recognition of nucleic acids in mammalian cells. The TLRs, RIG-1, MDA5, cGAS, AIM2, IFI16, and DDX41 are among the host restriction factors recognizing foreign DNA and RNA species. After binding to these restriction factors of the innate immunity system, through adaptor molecules and signal transduction, IRF3/7, Nf-kB, and AP-1 transcription factors become phosphorylated and migrate to the nucleus where type I IFNs and pro-inflammatory cytokines are expressed. RIG-1- retinoic acid-inducible gene I; MDA5-melanoma differentiation-associated protein 5; cGAS-cyclic GMP-AMP synthase; AIM2-absent in melanoma 2 protein; IFI16-interferon gamma inducible protein 16; DDX41- DEAD-box helicase 41; IRF3/7-interferon regulatory factor 3 or 7; Nf-kB-nuclear factor kappa B; AP-1-activator protein 1. Figure created with BioRender.com (accessed on 20 January 23).

Innate immunity is an evolutionary conserved system where cells contain an arsenal of specialized receptors, PRRs. Nucleic acids, are major ligands detected by PRRs (Figure 1). Innate immunity evolved to detect the invasion of pathogens and initiate host antimicrobial responses. However, it may also detect gene therapy vectors, hindering its efficacy or even eliciting unwanted side effects, such as the production of type I interferons and pro-inflammatory cytokines which lead to inflammation and tissue damage [5]. Once the gene therapy vectors are detected as invaders, inhibitory host RFs are up-regulated by interferons (IFNs). RFs can directly target evolutionarily conserved structural features on the vectors or exert broad and more indirect actions, as limiting the availability of cellular resources, such as nucleotides, transcription factors, or other factors [3]. Cyclic GMP-AMP synthase (cGAS) is an example of one such RF that acts indirectly; detects cytosolic double-stranded DNA (dsDNA), and dimerizes and triggers a cascade leading to type I IFN induction. IFNs are potent pleiotropic cytokines that broadly alter cellular functions, including changes in protein synthesis, proliferation, membrane composition, and nutritional microenvironment [6]. Intrinsic antiviral immunity refers to a form of innate immunity that directly restricts viral replication and assembly by rendering a cell non-permissive to a specific virus. Intrinsic immunity is conferred by RFs that are typically pre-existent in certain cell types. Intrinsic RFs recognize specific viral components, but unlike PRRs which inhibit viral infection indirectly by inducing interferons and other antiviral molecules, intrinsic antiviral factors block viral replication immediately and directly [7]. For example, the cytidine deaminases apolipoprotein B mRNA-editing enzyme catalytic polypeptide (APOBEC) edits the lentivirus’s genome during reverse transcription, converting cytidines to uridines that generate hypermutated defective viral genomes [7].

PRRs that recognize nucleic acids in cells can be generally divided in two groups based on their subcellular localization and expression patterns (Figure 1). The first group includes endosomal members of the Toll-like receptor family (TLR) that are present typically in immune cells. TLRs localize in the endosomal membrane and detect several forms of nucleic acid. They can also be at the cell membrane and translocate to the endosome. The second group of receptors is localized in the cytosol of almost all cell types. These include DNA receptors and RIG-I-like receptor (RLR) family members detecting RNA [8].

PAMPs (e.g., nucleic acids, liposacharides, peptidoglycans, proteins) are conserved molecular patterns that can be present in viral and non-viral vectors. Indeed, innate immune responses were also observed when using non-viral vectors. For example, the systemic injection of plasmid DNA with cationic lipids has been shown to elicit immune responses with tissue damage [9,10,11,12]. The same was observed when administering cationic polymer/plasmid DNA complexes [13]. Immune responses elicited by viral vectors based on DNA viruses, as adenoviral and AAV vectors, have also been documented [5,14,15,16,17]. RNAs are also potent activators of the innate immune system either delivered via non-viral or viral vectors. Indeed, exogenous mRNA is considered immunostimulatory, thus being explored in the development of vaccines [18]. Many interference RNA studies have also reported innate immune stimulation, attributed to features in the siRNA structure, sequence, and delivery mode [19]. RNA can activate innate immunity through TLRs, blocking RNA translation, and promoting mRNA degradation. In non-immune cells, the cytoplasmic receptors sense RNA and mediate cytokine and chemokine production [20].

RFs are therefore major bottlenecks to overcome in both viral and non-viral gene transfer applications. Here, we review recent advances elucidating the mechanism by which RFs impact the transduction efficiency of AAV and lentiviral vectors. 

## 2. Lentiviral Vectors

**Figure 2:**
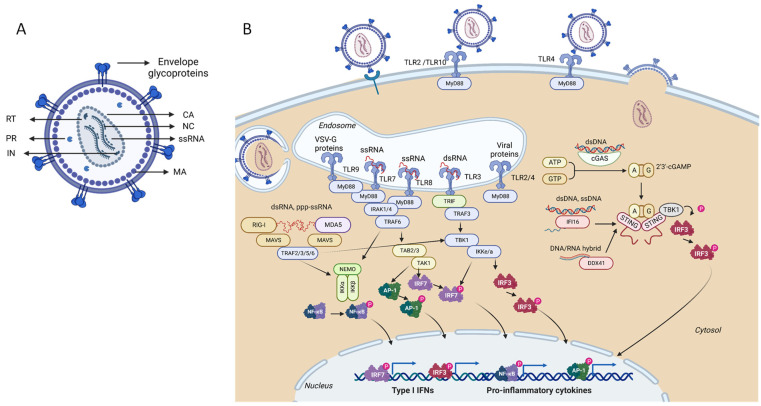
Schematic representation of lentiviral vector particles depicting its composition: (**A**) NC, nucleocapsid; MA, matrix; CA, capsid; RT, reverse transcriptase; PR, protease; IN, integrase; ssRNA, single-stranded RNA lentiviral vector genome. Pattern recognition receptors sensing lentiviral vectors and innate immunity; (**B**) During lentiviral vector transduction, the host cell is exposed to viral envelope antigens, genomic, ssRNA, reverse transcription intermediates, viral capsid, and integrated therapeutic cassette. Recognition of envelope antigen can occur via TLR2, TLR4, or TLR10 and leads to NF-kB-mediated inflammatory responses. Recognition of genomic ssRNA via endosomal TLR7 or TLR8 induces NF-kB-dependent inflammatory responses, IRF7-dependent interferon responses, or AP-1-interferon response, depending on the cell type. Recognition of ssRNA and dsRNA by RIG-I induces IRF3-dependent interferon responses and Nf-kB -dependent inflammatory cytokines. Recognition of HIV-1 capsid can be mediated via Cyclophilin A and TRIM5, resulting in IRF3-STING dependent interferon responses and NF-kB-dependent inflammatory responses. Recognition of dsDNA by cGAS, IFI16, or DDX41 sensor induces the interferon response via the STING-IRF3 axis. Adapter molecules and PAMPs are merely indicative and may change depending on the cell type and the host cell environmental. DNA/RNA represent hybrid molecules. TRIM5-tripartite motif-containing protein 5; STING-stimulator of interferon genes. Figure created with BioRender.com (accessed on 15 December 2022).

Lentiviral vectors are derived from lentiviruses that belong to the Retroviridae family of complex retroviruses. Retroviruses are RNA-enveloped viruses with the ability to “reverse transcribe” their genome from RNA to DNA, a step that occurs in the cytoplasm of host cells [21]. Lentiviruses are particles of approximately 80–120 nm and contain two identical copies of linear single-stranded, positive-sense RNA (Figure 2A). The genome is complexed with nucleocapsid proteins. The virus codes for three enzymes: reverse transcriptase, integrase, and a protease. The ssRNA is contained in the nucleocapsid, the inner portion of the virus. The nucleocapsid is enclosed by a protein shell formed by capsid proteins. Matrix proteins form a layer outside the capsid and interact with the envelope lipid bilayer surrounding the viral core. The envelope is derived from the cell host membrane and incorporates the viral envelope glycoproteins responsible for the viral particle binding to specific cell receptors [21,22]. In addition to the enzymes and structural proteins mentioned above, coded by the *gag-pol* and *envelope* genes, human lentiviruses also code for two regulatory proteins (Tat and Rev) and four accessory proteins: Vif, Nef, Vpr, and Vpu [21].

LV vectors can be derived from different lentivirus species (e.g., *Simian immunodeficiency virus*, *Bovine immunodeficiency virus*, *Equine infectious anemia virus*, and *Feline immunodeficiency virus*) but the most used are based on *Human immunodeficiency virus 1* (HIV-1) that will be the primary focus of this review. HIV-1 LV vectors only code for the therapeutic gene of interest (GOI). Therefore, lentiviral vector RNA genome, in addition to a heterologous promotor and the GOI, only retains, from the wild-type viruses, the cis-acting sequences which are required for its packaging, its reverse transcription, transport into the cellular nucleus and, integration into the cell genome [22]. The LV vector particles contain only structural and enzymatic proteins. Accessory proteins are removed from the second LV vector generation onwards. Regulatory proteins are not incorporated into virion particles. The envelope protein most used to generate lentiviral vectors is the vesicular stomatitis virus G protein (VSV-G), due to its efficient pseudotyping and broad tropism, but others can be used [23]. The route of entry of LV vectors is determined by the envelope glycoprotein. VSV-G pseudotypes exhibit a pH-dependent entry mechanism. After receptor attachment vector particles enter the cell via endocytosis [24]. Other envelopes, such as those derived from gamma-retroviruses, use a pH-independent entry mechanism and, after receptor binding, enter the cell directly by fusion with the plasma membrane [24]. In both entry pathways, the capsid is liberated and disassembled into the cytoplasm.

Currently, LV vectors are the vector of choice to perform ex vivo gene transfer to hematopoietic stem cells, to treat inherited genetic diseases, and T and natural killer (NK) cells for immunotherapies, namely T cell receptor (TCR) and chimeric antigen receptor (CAR) therapies [25,26]. The development of in vivo LV vector-based therapies extends from applications targeting the central nervous system (CNS), targeting neurons or glial cells (e.g., neurodegenerative or lysosomal storage diseases), to liver-directed gene therapy (e.g., correction of hemophilia) [27,28,29].

### 2.1. Innate Immunity and Antiviral Restriction Factors

Pre-existing immunity to LV vectors in humans is low, an advantage of these vectors, but during the several steps of vector transduction, LV vectors nucleic acids and proteins can be recognized by RFs acting as innate immunity sensors [30]. Lentiviral vectors rely mostly on the same viral and cellular machinery as HIV-1 to reach the nucleus and integrate their genome on target cells. As such, they are susceptible to most RFs identified in the context of HIV-1 wild-type virus infection.

RFs are host cellular proteins that recognize and interfere with specific steps of the replication cycle of viruses, thereby blocking infection. These can have constitutive expression in different cell types, or be IFNs or DAMPs inducible, but generally their inherent features, such as their self-sufficient activity and rapidity of action, confer early restriction to viruses [31].

Several restriction factors have been identified to target the retroviral life cycle. However, while some of those are ubiquitously expressed in mammalian cells, others are cell-specific or only expressed upon induction. This explains why some cell types are efficiently transduced, while others poorly, by LV vectors [32]. The mode of delivery and the envelope glycoprotein used in LV pseudotyping will also impact the immune response.

Here, we review and discuss the innate immune response through PRRs and intrinsic antiviral restriction factors and how these hamper successful LV vector transduction and clinic efficacy. The cells where each RF expression was observed are indicated.

#### 2.1.1. Restriction Factors Sensing Lentiviral Vector RNA

Lentiviral vectors have two strands of genomic ssRNA encapsulated. These nucleic acids, as well as their transcripts, can potentially act as PAMPs recognizable by a PRRs and initiating a cascade of innate signaling, leading to the induction of the host’s anti-viral response.

Both Toll-like receptors (TLRs) and RIG-I-like receptors, family members of PRRs, have been identified as sensors of HIV-1 RNAs (Figure 2B and Table 1) [33]. TLR 7 and 8 detect ssRNA and TLR3 dsRNA species from lentivirus. TLR7, and 8 can recognize ssRNA in incoming retroviruses in the endosomal compartment. This recognition does not require viral replication [34,35,36]. Further studies indicate that TLR7 preferentially recognizes the guanosine (G)- and uridine (U)-rich ssRNA oligonucleotides [37].

MyD88 is the main adaptor molecule responsible for the activation of the innate response pathway for all TLRs with the exception of the TLR3 [38]. The MyD88 pathway is predominantly involved in the production of inflammatory cytokines and IFN type I through the translocation of NF-kB to the nucleus. TL3 uses the TIR-domain-containing adapter-inducing interferon-β (TRIF), i.e., the TRIF adaptor molecule will act through interferon regulatory factor (IRF) 3 and 7, leading to the production of IFNs type I [38]. TLR7 is primarily expressed in plasmacytoid dendritic cells, while TLR8 on myeloid cells (including dendritic cells, monocytes, and macrophages). The activation of those TLR pathways in LV transduction have been mostly observed in dendritic cells (DCs) [39]. Some authors have suggested that since TLR3, 7, and 8 are endosomal, that some LV pseudotypes may escape from their detection in the endosomes, particularly, LV pseudotypes that can enter via direct fusion with the cytoplasmic membrane [32]. However, entry routes are not always completely elucidated and the multiple PRR sensors detecting infection at different stages of the viral cycle complicate the direct validation of this hypothesis.

RIG-I and its homolog melanoma-differentiation-associated protein 5 (MDA5) are RNA helicases localized in the cytoplasm, and were reported to detect dimeric and monomeric RNAs of HIV-1 in infected macrophages [23,40]. RIG-I-like receptor family, initially identified as DExD/H-box-containing proteins, comprise RIG-I, MDA5, and LGP2 (laboratory of genetics and physiology 2) and share highly conserved domain structures: a central DExD/H box helicase core and a C-terminal domain (CTD) that confers part of the ligand specificity. Both RIG-I and MDA5 have caspase activation and recruitment domains (CARD) mediating signaling to downstream adaptor proteins [8].

RIG-I detects RNAs possessing an uncapped 5′-di/triphosphate end (5′ ppp ssRNA) and a short blunt-ended double-strand portion, two essential features facilitating the discrimination of viral from self-RNAs [41]. The preferential binding of RNAs to RIG-I and MDA5 is attributed to the fragment length, long fragments (˃ 4kb) are preferred by the latter, while shorter ones are preferred by the former [8]. After binding to stimulatory RNAs, RIG-I and MDA5 RLRs go through a ATP-dependent conformational change enabling binding to downstream adapter molecules through the oligomeric assembly of the CARD domain [42]. Mitochondrial antiviral signaling protein (MAVS), also containing a CARD domain, is activated by RIG-I and MDA5 oligomers. MAVS aggregation on the surface of mitochondria and the formation of protein filaments via CARDS initiates downstream signaling. Aggregated MAVS recruit and activate E3 ligases TRAF2,3,5 and 6 which synthesize polyubiquitin that are sensed by NEMO proteins that recruit IKK and TBK1 proteins. The latter two, phosphorylate IRF3, 7 and IkB, lead to the transcription of type I IFNs and pro-inflammatory cytokines (e.g., TNFα, IL-6, IL-8, IL-1B, INF). NfkB is also activated via the IKK complex.

HIV-1 RNAs are, however, enclosed within the capsid core, likely shielded from the innate immune sensors. The uncoating of the capsid occurs upon the initiation of reverse transcription, resulting in viral RNA exposure, although minimal, to RIG-I [33,43,44]. Therefore, RIG-I mediated signaling may play a limited role in triggering antiviral immunity during the early steps of LV vector transduction [33].

#### 2.1.2. Restriction Factors Sensing Lentiviral Vector Reverse Transcription Products

HIV-1 reverse transcription intermediates (e.g., cDNA, ssDNA, and DNA/RNA hybrids) can be sensed by DNA PRRs as interferon gamma inducible protein 16 (IFI16), cyclic GMP-AMP synthase (cGAS), and helicase DDX41 [33].

IFI16 is a HIV-1 DNA species sensor in macrophages and tonsillar CD4+ T cells [40,45]. It detects incomplete HIV-1 DNA reverse transcripts accumulated in the cytoplasm of abortively infected tonsillar lymphoid cells. It is believed that upon binding to HIV-1 cDNA, IFI16 recruits a stimulator of interferon genes (STING) to activate the TANK-binding kinase 1 (TBK1) and IRF3 signaling axis, resulting in the transcription of antiviral genes in myeloid cells. In tonsillar lymphoid cells, IFI16 activates the inflammasome pathway through ASC and caspase-1, leading to IL-1β production [40,45]. This may indicate that IFI16 is cell-type dependent [33,46]. IFI16 recognizes abortive RT products to induce IFNs, as well as inflammasome activation by binding to the adapter molecule ASC (apoptosis-associated speck-like protein containing a CARD). This leads to the activation of caspase-1 and cytokine IL-1β, triggering pyroptosis.

During reverse transcription, DNA/RNA hybrids are created. DDX41, an RNA helicase protein thought to function in RNA splicing, was recently identified as the sensor that primarily binds the short-lived murine leukemia virus (MLV) DNA/RNA hybrids. Its binding to DNA/RNA hybrids activates downstream signals in a STING-dependent manner in primary mouse macrophages and DCs [40]. 

cGAS is widely recognized as the major sensor to HIV-1 DNA [47]. cGAS preferentially recognizes the stem-loop structures of single-stranded DNA (ssDNA), derived from HIV-1 cDNA. The DNA damage induced by HIV-1 integration might also be linked to cGAS-mediated signaling activation. cGAS might be responsible for the innate immune sensing of HIV-1 integration by being drafted to the nucleus and recognizing self-DNA from damaged chromatin. cGas is thus recruited to the double-stranded breaks to suppress homologous recombination [48]. Notwithstanding, cGas is negatively regulated by cellular proteins to avoid auto-immune responses. cGAS responds to threshold levels of accumulated viral DNA, thus avoiding aberrant activation by self-DNA species. Sterile alpha motif and histidine-aspartate domain-containing protein 1 (SAMHD1) and TREX1 (three prime repair exonuclease 1), are host factors limiting the accumulation of DNA that can be sensed by cGAS in the cytoplasm [49,50]. SAMHD1 prevents the accumulation of ssDNA by promoting the degradation of nascent DNA at stalled replication forks, which may escape the nucleus during mitosis, thereby limiting innate immune sensing by cGAS [51]. It has also been reported that SAMHD1 interacts with the inhibitor-κB kinase ε (IKKε) and IRF7 to suppress the innate immune response by reducing IKKε-mediated IRF7 phosphorylation [52,53]. TREX1 is a ubiquitously expressed exonuclease that prevents the activation of the cGAS/STING/IRF3 signaling axis by eliminating cytoplasmic DNAs before their detection by cGAS [53,54]. cGas antiviral response is generated by the STING-TBK1-IRF3 axis, i.e., upon stimulation, cGas synthetizes cyclic-di-GMP-AMP (cGAMP), that binds and activates the endoplasmic reticulum adaptor STING. STING engages the TANK-binding kinase 1 (TBK1) complex, activating IRF3 by phosphorylation leading to the expression of IFN type 1 (Figure 2B).

#### 2.1.3. Restriction Factors Sensing Lentiviral Vector Proteins

TLRs expressed on cell membranes can recognize virus PAMPs and activate innate immune responses. The cell surface-expressed TLR2 and TLR4 have been reported in sensing HIV-1 glycoprotein gp120, in particular, in mucosal epithelial cells, leading to the activation of NF-kB and production of inflammatory cytokines [48]. Soluble TLR2, i.e., sTLR2 was first detected in milk and later shown to act as an inhibitor of virus entry [55]. Henrick et al. showed that TLR2 binds to gp41, core protein p24, and matrix protein p17, resulting in the inhibition of NF-κB activation [56]. It was also observed that the HIV-1 co-receptor CCR5 is highly downregulated through the TLR2-dependent pathway arresting infection [57]. 

Recently, HIV-1 gp41 was identified to be recognized by the TLR10 ligand in MCF-10A and THP-1 cells, leading to the generation of IL-8 and NF-kB activation. However, the exact mechanism is not known, and it cannot be ruled out that spontaneous heterodimerization of TLR2 and TLR10 occurs due to HIV-1 gp41 (acting as a common ligand for both TLRs) [58]. Information on VSV-G glycoprotein recognition by PRRs is scarce. However, it has been proposed that VSV-G pseudotyped LV vectors form tubulovesicular structures with DNA fragments, promoting TLR9-signaling structures [59].

In addition to the PRRs that recognize envelope glycoproteins, RFs sensing the HIV-1 capsid were identified [33]. While some host factors are hijacked by the HIV-1 capsid to prevent the innate sensing of HIV-1 infection (e.g., cyclophilin A (CypA) and specificity factor subunit 6 (CPSF6)), others, such as tripartite motif-containing protein 5 (TRIM5) and NONO, activate innate immune signaling [33]. These factors are, however, in the category of intrinsic antiviral restriction factors and will be described in the following section.

#### 2.1.4. Lentiviral Vector Intrinsic Restriction Factors

The antiviral actions exerted by intrinsic RFs are immediate and direct and are herein defined as ‘intrinsic antiviral immunity’ to distinguish from the ones requiring effectors induced by IFNs and cytokines through transcriptional activation. Intrinsic viral restriction factors are usually preexisting in certain cell types, rendering the cell non-permissive to certain viruses. Notwithstanding, intrinsic RFs are frequently IFN-inducible, i.e., are interferon stimulated genes (ISGs), linking intrinsic and innate immunity. The production of IFNs will therefore stimulate their expression activation and upregulation. 

Examples of intrinsic restriction factors are interferon-inducible transmembrane (IFITMS), tripartite motif containing 5 (TRIM5) proteins, the human apolipoprotein B mRNA-editing enzyme, catalytic polypeptide-like 3 (APOBEC3) family, myxovirus resistance protein 2 (Mx2 or MxB), SAMHD1, and bone marrow stromal antigen 2 BST/tetherin/BST-2. The latter are briefly described below.

IFITMs are a family of small proteins of type II transmembrane proteins. IFITM1, IFITM2, and IFITM3 are expressed almost ubiquitously in humans, whereas IFITM5 is primarily expressed in osteoblasts [60]. IFITM1, IFITM2, IFITM3 genes are ISGs with an interferon-stimulated response element (ISRE) in their promoter region [61]. IFITM5 and IFITM10 are not induced by IFNs [60]. IFITMs restrict a great number of enveloped RNA viruses. IFITM-mediated antiviral activity spans from the inhibition of viral entry to the inhibition of viral protein synthesis [62]. IFITMs that localize at the plasma membrane, as well as at the membranes of endocytic vesicles and lysosomes, restrict viral infections by inhibiting virus entry [63]. IFITMs block viral entry by impairing the hemifusion process, most likely by reducing membrane fluidity. Although the first antiviral action of IFITMs is to protect target cells from incoming viruses, a second antiviral mechanism of action is the production of virions that package IFITMs. The latter virions display a reduced entry capacity; this was observed for HIV-1, thus the absence of IFITMs in LV packaging cells is essential. Yu and colleagues explain the impairment of lentivirus fusogenic capacity by the decrease in the number of envelope spikes in the particles [64]. IFITM2 and IFITM3 avert viral entry, and IFITM1, IFITM2, and IFITM3 prevent Gag production [65]. 

TRIM proteins constitute a large family of E3 ubiquitin ligases involved in many cellular functions, such as cell differentiation, apoptosis, autophagy, immunity, and antiviral functions [60]. The natural functions of TRIM5α are not totally known, but it is one of the best studied, species-specific barriers in lentiviral transduction because it restricts lentivirus by uncoating viral particles, while activating early innate responses [38]. Several TRIM proteins have antiretroviral activity, and TRIM5α was identified as the factor responsible for HIV-1 restriction [65]. TRIM5α is a cytoplasmic protein ubiquitously expressed in all tissues in the human body, and although expression levels may be constitutively low, they can be upregulated by IFN through a putative ISRE [66]. Restriction patterns are variable between species, e.g., TRIM5α from the rhesus macaque strongly inhibits HIV-1, but not SIV, whereas human TRIM5α restricts equine infectious anemia virus. Human TRIM5α also mediates a mild restriction of HIV-2, but not as high as to HIV-1 [66]. The species specificities are attributed to CA sequence differences between viruses and subsequently the TRIM5α ability to recognize and bind. Although Ribeiro and colleagues have shown that TRIM5α can inhibit HIV-1 infection in some DC subsets, this antiviral function depends on the route of HIV-1 internalization [67]. 

TRIM5 recognizes capsids when in combination with cyclophilin A (CypA) and degrades LV vectors by binding and destabilizing the capsid, preventing reverse transcription completion [68]. TRIM5α leads to the accelerated and disrupted uncoating of the virus [66]. The TRIM5α E3 ubiquitin ligase RING domain has an effector function, recruiting proteasomal machinery. Proteasomes inhibition has shown to prevent the premature disassembly of the capsid and restores HIV-1 reverse transcription [60]. Human CD34+ CD38- cells have high TRIM5 expression, which may explain the lower LV vector transduction efficiency of hematopoietic stem/progenitor cells (HSPCs), which also varies among individuals [69]. TRIM5α expression levels are negatively correlated with LV vector transduction efficiency in human T-lymphocyte cell lines and CD34+ cells [70]. The treatment of HeLa and HepG2 cells with IFN-I increases TRIM5α mRNA levels [71]. A systematic analysis of TRIM gene expression in human primary lymphocytes and monocyte-derived macrophages in response to IFN-I and IFN-II has revealed that several TRIM genes are upregulated by IFNs. Among the 72 human TRIM genes tested, 27 were sensitive to IFN, including TRIM5α [72].

The human APOBEC cytidine deaminases family is well described as potent and well-characterized HIV RFs. APOBEC3G was the first restriction factor identified for HIV-1. Vif mediates the proteasomal degradation of APOBEC3G, counteracting its antiviral function. The 2nd and 3rd generation of LV vectors do not have Vif making them susceptible to APOBEC3G. APOBEC3 proteins can co-package into the LV particles [73]. Thus, it is important to verify that no APOBEC3 protein is expressed during LV vector production to avoid mutations. APOBEC3G is incorporated into the core of Vif-deficient particles through interactions with the nucleocapsid and viral RNA [74]. Once the particles infect a new cell, APOBEC3G remains associated with the mature viral proteins and RNA. 

APOBEC3G enzymatic activity is capable of mutating DNA by cytidine deamination during reverse transcription. The catalyzed cytosine-to-uracil deamination in the nascent viral DNA lead to a high frequency of G-to-A hypermutations introducing amino acid substitutions and premature STOP codons [31,66]. Additionally, APOBEC3 can act without the deaminase activity for its antiviral activity. It was observed in several studies that it can block reverse transcription elongation in a deaminase-independent manner It was observed in several studies that it can block reverse transcription elongation in a deaminase-independent manner [75].

APOBEC3G genes are most abundant in monocytes, macro-phages, DCs, resting CD4+ T cells but not in activated CD4+ T cells. The activation of innate immunity, in particular by the I IFN response, increases APOBEC3G expression. The treatment of immature DCs or monocyte-derived macrophages, with synthetic TLR3 ligands, leads to APOBEC3G expression and induces antiviral activity against HIV-1 [76,77]. The stimulation of human monocyte-derived dendritic cells with Gag virus-like particles, which are recognized by PRRs, generates an increase in the mRNA and protein expression of APOBEC3G [78]. Furthermore, several cytokines involved in innate and adaptive immunity, such as IL-2, IL-7, and IL-15, induce APOBEC3G in peripheral blood lymphocytes [79]. 

MX2 is a protein belonging to the dynamin superfamily of large guanosine triphosphatases (GTPases) [80]. MX2 acts after the reverse transcription step. It localizes in the cytoplasmic side of nuclear pores and regulates the nucleocytoplasmic transport of viral DNA. Borsoti et al. hypothesize that MX2 activity impacts LV vector nuclear translocation in transduced cells [38]. The N-terminal 25 amino acids of MX2 interact with HIV-1 CA, leading to the abrogation of HIV-1 infection [62]. Some studies suggest that Mx2 can impair HIV uncoating by binding to the capsid [81]. Mx2 may affect nuclear entry or post-nuclear trafficking without destabilizing the inherent catalytic activity of viral pre-integration complexes. The interaction between Mx2 and the viral capsid is either direct or mediated via CypA [82,83]. Mx2 GTPase activity is required for oligomer assembly, required for its antiviral function; however, the activity is dispensable for its restriction activity [60]. Buffone and colleagues showed that Mx2 could be involved in HIV restriction through interaction with SAMHD1 [84]. Mx2 is part of the ISG family and is induced by IFN-α and IFN-β, as shown in human fibroblasts [85]. After IFN treatment, Mx2 proteins were detected in human primary monocytes, lymphocytes, and macrophages [60]. 

SAMHD1 is a deoxynucleoside triphosphate phosphohydrolase (dNTPase). It hydrolyzes all four dNTPs, impairing HIV reverse transcription by decreasing the pool of nucleotides available for reverse transcription [60]. Recent data shows that it also has RNase activity that seems to be important for HIV restriction. SAMHD1 can bind ssDNA and RNA being able to degrade RNA–DNA duplexes and HIV genomic RNA [86,87,88]. Vpx, encoded by HIV-2 and SIV variants, but not HIV-1, targets SAMHD1 for proteasomal degradation (observed in myeloid and resting CD4+ T cells) [38].

SAMHD1 is not an ISG; its expression is not induced by IFN-I in DCs and primary CD4+ T cells. However, in some human cells lines (HeLa, HEK293, and MARC-145 cells), the activations of TLR3 and RIG-I/MDA5 upregulated SAMHD1 expression through IRF3 [60,89]. As SAMHD1 is regulated at the post-transcriptional level, and in a cell-cycle-dependent manner, it has low antiviral efficiency in dividing cells. In liver cells, SAMDH1 expression is only observed upon IFN-α treatment via STAT1-, STAT2-, and IRF9-dependent pathway [90]. SAMDH1 constitutive expression is missing in T-cell lines [60]. The constitutive expression of SAMHD1 is observed in cells of the myeloid lineage, inhibiting HIV-1 replication in DCs, monocytes, and macrophages, as well as in CD4+ T cells. IL-12 and IL-18 treatments in monocyte-derived macrophages lead to SAMHD1 overexpression [90]. HIV-1 infection is restricted by SAMHD1 in resting CD4+ T cells but not in activated T cells [91,92]. The stimulation of resting CD4+ T cells with IL-7 induces T592 phosphorylation of SAMHD1 and abrogates its antiviral activity [93]. The high SAMHD1 expression in HSPCs explains the resistance of HSCs to LV vectors [38]. While in activated CD4+ T cells, the high expression of SAMHD1 corresponds to a high concentration of dNTPs and a high permissiveness to HIV-1-based transduction. In HSPCs, an entirely different situation was shown with a low concentration of dNTPs and a low permissiveness to HIV-1-based LVs [38,94,95]. SAMHD1 is also expressed in B cells [94] which are resistant to LV transduction. However, by the use of different envelope pseudotypes, it was possible to remove the block on both B cells and HSPCs [96]. Thus, LV vector envelope glycoprotein pseudotyping seems to impact vector permissiveness.

Tetherin, or BST2, belongs to the ISGs and was identified as a RF in HIV-1 infection. HIV-1 can escape restriction imposed by tetherin through Vpu but LV vectors do not contain the latter [38]. Tetherin is a dimeric Type II membrane protein with an N-terminal cytoplasmic tail, a transmembrane region, and a C-terminal glycophosphatidyl inositol anchor. Within the short cytoplasmic tail, tetherin encodes a dual-tyrosine motif important for clathrin-mediated endocytosis. In vitro cell culture studies revealed that HIV-1 Gag co-localizes with tetherin in intracellular compartments, suggesting that the internalization of tetherin-bound virions could lead to its degradation through the endolysosomal pathway [97]. Thus, the most consensual mode of action attributed to tetherin is the entrapment of viral particles at the surface of host cells preventing their release. This was observed in vitro [98]. To note, however, that other modes of action have been attributed to tetherin, namely, an immunomodulatory role, which may have positive or negative influence on viral replication. In the context of LV vectors, tetherin seems to be a concern when expressed in the producer cells.

Tetherin is expressed at different levels in several human tissues. It is highly expressed on blood vessels [60]. The promoter region of the tetherin gene has a binding site for the transcription factor STAT3, suggesting it can be upregulated by innate immune-signaling pathways, such as IFN-I. Tetherin expression was observed to be upregulated in human umbilical vein endothelial cells, by the three types of IFNs (IFN-α, IFN-γ, and IFN-λ), in hepatocytes by IFN-I, and in neurons via a STAT1-dependent pathway [99,100]. IFN-α and TLR3 or TLR4 engagement also upregulates tetherin in myeloid DCs, monocyte-derived DCs, and macrophages [77,101]. Altogether, this data supports tetherin gene-expression induction by innate immunity after the sensing of infection by TLRs. This is confirmed in vivo studies showing that HIV infection upregulates its expression in human peripheral blood mononuclear cells.

The full extent of activity and regulation of the above-described RFs is still to be elucidated. Additionally, there are hundreds of other RF antiviral proteins. Examples of these are: protein kinase R (PKR), the 2′-5′-oligoadenylate synthetase 1 (OAS1) schlafen protein 11 (SLFN11), DEAD box helicases (DDX) 3X (DDX3X), cholesterol 25-hydroxylase (CH25H), interferon-stimulated gene 15 (ISG15), and non-POU domain-containing octamer-binding protein (NONO). Most of the intrinsic RFs are IFN-inducible, linking intrinsic and innate immunity and making their study complex.

**Table 1:** Host restriction factors involved in innate sensing HIV-1.

Viral Infection Phase	Sensor	Ligands	Adaptors	Effectors
Entry/ Uncoating	TLR2/4/10	Viral proteins	MyD88	Inflammatory cytokines/Type I IFNs
	TLR3	dsRNA	TRIF	Inflammatory cytokines/Type I IFNs
	TLR7	ssRNA	MyD88	Inflammatory cytokines/Type I IFNs
	TLR8	ssRNA	MyD88	Inflammatory cytokines/Type I IFNs
	TLR9	CpG DNA Viral proteins	MyD88	Inflammatory cytokines/Type I IFNs
	RIG-I/MDA5	dsRNA, ppp-sRNA	MAVS	Inflammatory cytokines/Type I IFNs
	IFITMS	Viral envelope	d.a.	Abortive infection
Uncoating/ Reverse Transcription	cGas	dsDNA	STING	Inflammatory cytokines/Type I IFNs
	IFI16	DNA/RNA	STING/ASC	Inflammatory cytokines/Type I IFNs Caspase-1
	DDX41	dsDNA, ssDNA	STING	IFNs
	TRIM5	CA	d.a.	Abortive infection
	APOBEC3	NC/ssRNA	d.a.	Abortive infection
	SAMDH1	ssDNA, ssRNA, DNA/RNA	d.a.	Abortive infection
Integration	cGas	DAMP response	STING	IFNs
	Mx2	CA	d.a.	Abortive infection
Transcription/ Translation	DDX3X	Abortive viral RNA	MAVS	IFNs
Other	Tetherin	CA	d.a.	Abortive infection Inflammatory cytokines/Type I IFNs

d.a., direct actuators

## 3. Adeno-Associated Vectors

Adeno-associated virus (AAV) belongs to the Parvoviridae family and its life cycle is dependent on the presence of a helper virus. Thus, AAV are replication-defective. The current consensus is that AAV does not cause any human diseases [102]. 

The AAV viral particles are composed of an icosahedral protein capsid of approximately 26 nm in diameter and a single-stranded DNA genome of approximately 4.7 kb that can be either a plus (sense) or a minus (anti-sense) strand. [103]. The genome is flanked by two T-shaped inverted terminal repeats (ITRs) at the ends that serve as viral origins for replication and packaging signal [102]. The AAV genome encodes three genes. The Cap gene encodes the three VP proteins through alternative splicing and translation from different start codons. The Rep gene codes for the four proteins required for viral replication. These, named after their molecular masses, are Rep78, Rep68, Rep52, and Rep40. The third gene generates the assembly activating protein (AAP) and is encoded within the cap coding sequence in a different reading frame. AAP promotes virion assembly [104]. AAV2 can integrate into a genomic locus denominated AAVS1 in human cells to establish latency [105], a phenomenon mediated by Rep activity.

**Figure 3:**
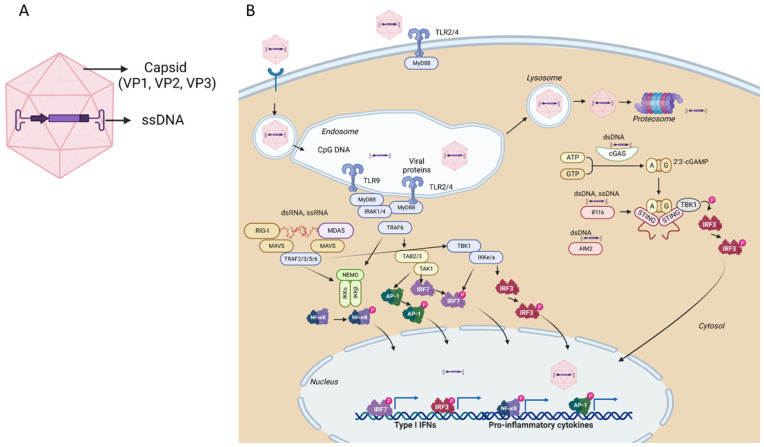
Schematic representation of an adeno-associated virus vector particles depicting its composition: (**A**) VP, viral protein (VP1, VP2, and VP3 constitute the viral capsid); ssDNA, single-stranded DNA AAV vector genome. Pattern recognition receptors sensing AAV vectors and innate immunity; (**B**) During AAV vector transduction, the host cell is exposed to viral proteins, viral genomic ssDNA, and transcripts generated from the ITRs. Capsid proteins can potentially be recognized by TLR2 and 4, particularly if they contain post-translational modifications, leading to NF-kB-mediated inflammatory responses. After AAV entry, by endocytosis, AAV capsids can be degraded in endosomes, causing the AAV genome or capsid to be exposed to receptors, such as TLR9, TLR2, and TLR4, triggering an innate immune response via a MyD88-driven pathway and NF-kB-mediated inflammatory response. AAV virions that successfully escape from endosomes can have their capsids ubiquitylated and SUMOylated in the cytoplasm and are targeted for degradation. This exposes the AAV genome to cGas, IFI16, and AIM2, resulting in IRF3-STING dependent interferon responses and NF-kB-dependent inflammatory responses. AAV vectors that reach the nucleus and uncoat may proceed to transcription where plus-stranded and minus-stranded RNAs are generated from both ITRs of the AAV cassette. The latter are exported to the cytoplasm to form double-stranded RNA (dsRNA) that can be recognized by the dsRNA sensors MDA5 and RIG-I and induces IRF3-dependent interferon responses and Nf-kb inflammatory cytokines. Adapter molecules and PAMPs are merely indicative and may change depending on the cell type and the host cell environment. DNA/RNA represent hybrid molecules. Figure created with BioRender.com (accessed on 15 December 2022).

AAV vectors used for gene therapy are composed of the same capsid sequence and structure as found in wild-type AAVs; however, the genomes are devoid of all viral protein-coding sequences and just express the therapeutic gene of interest (Figure 3A). The only sequences of viral origin that remain are the ITRs, which are needed to guide genome replication and packaging during vector production [106]. The complete removal of viral coding sequences, namely, Rep genes, renders genome integration greatly reduced. Thirteen natural serotypes of AAV have been isolated and more than 100 variants have been explored in the context of gene therapy. The AAV capsid can accommodate mutations and ligand insertions, enabling the generation of capsid-engineered variants [107]. The capsid is responsible for the vector tropism and tissue specificity.

Currently, AAV vectors are the vector of choice to perform in vivo gene transfer to several tissues to treat inherited genetic diseases. The development of AAV vector-based therapies in the clinic extend from broad therapeutic areas as blood disorders (e.g., correction of hemophilia through liver-directed gene therapy), central nervous system diseases (e.g., Parkinson’s), eye disorders (e.g., Leber congenital amaurosis), lysosomal storage disorders (e.g., GM1 gangliosidosis), and muscular and neuromuscular conditions (e.g., Duchenne muscular dystrophy and spinal muscular atrophy).

### 3.1. Innate Immunity and Antiviral Restriction Factors

AAV are non-pathogenic, thus, most of the knowledge related to innate immune response were obtained in the context of gene therapy applications. Due to the inherent nature of the AAV vector, a non-enveloped particle with repetitive capsid motifs, one of the major concerns is the adaptive immune response directed at AAV antigens. It is currently known that AAV wild-type infection prevalence among the human population is between 35 to 80%, depending on the serotype, making the unwanted adaptive response an important concern and side effect in AAV vector-mediated therapies [108,109]. However, the innate immune response stimulates the adaptive immune responses. The converse also occurs. The adaptive immunity against AAV vectors enhances the protective mechanisms of innate immunity, thus making it difficult to elucidate the primary cause of the immune responses observed. Antibodies against the AAV capsid will hamper vector transduction, therapeutic gene expression and overall gene therapy efficacy. 

Here, we review and discuss the innate immune response through PRRs and intrinsic antiviral restriction factors. RFs are the first cell barrier against AAV transduction, stimulating the adaptive immunity, and altogether hampering gene therapy efficacy. First, PRR restriction factors recognizing the AAV vector nucleic acid genome, their nucleic acids intermediate species and, proteins are described (Figure 3B). Thereafter, recent molecules suggested as intrinsic RFs against AAV vectors are reviewed (Table 2). The cells where each RF expression was observed are indicated.

#### 3.1.1. Restriction Factors Sensing AAV Vector DNA

AAV vectors have one genomic single-stranded (ss) DNA encapsulated. The AAV vector genome, as well as its transcripts can act as PAMPs recognizable by host PRRs to initiate a cascade of innate signaling, and the induction of the host anti-viral response. These PRRs, as discussed in the previous sections, are widely expressed in innate immune cells as dendritic cells, macrophages, B cells, and some T cells, but can also be found in some non-immune cells, such as fibroblasts and epithelial cells. Even when the primary target cell of therapy does not express high levels of PRRs the nature of in vivo delivery system and the high doses of vectors used in AAV-based therapies often lead to off-targeting of cells that express them. Ultimately, the release of IFNs will lead to the expression of ISG in the target cells.

AAV vectors attach the cells by binding to specific receptors, which may differ according to the serotype, and enter through endocytosis. After endocytosis, AAV particles traffic through the endosome, where capsid in the acidic environment extrudes VP1 unique portion, exposing the phospholipase domain to facilitate endosomal escape [110,111]. The trafficking of the AAV vector genome to the nucleus is still not fully elucidated, namely, where the capsid disassembly occurs. It is known that the capsid contains pores at the fivefold axis of symmetry where potentially the ssDNA could exit [112]. It is also well known that many AAV vectors do not reach the nucleus and suffer proteasomal degradation that increases the availability of AAV genomes for intracellular recognition [113].

Independently of the mechanism, capsid disassembly, escape through pores or proteasomal degradations, when the genome becomes exposed, it can be recognized by PRRs. TLR9, which recognizes CpG DNA from AAV vectors, will signal through MyD88 leading to the activation of Nf-kB and ISG expression. Several studies support this pathway activation in AAV vector transduction. Zhu and colleagues showed that AAV activates mouse and human plasmacytoid DCs via TLR9 to produce type I IFNs. TLR9 knockout plasmacytoid DCs fail to secrete IFN-α [114]. The authors also show that the activation of the TLR9-MyD88 pathway by AAV is independent of the nature of the transgene or AAV serotype. Martino et al. (2011) studied the innate immune response to self-complementary AAV vectors (scAAV) on hepatic gene transfer in mice [115]. For single-stranded AAV vectors a rapid, milder, and transient immune response was observed, up-regulating type 1 IFN, TLR9, MyD88, TNF-α expression in the liver. When using scAAV vectors, higher increases of those transcripts were observed after administration. Simultaneously, an upregulation of additional pro-inflammatory genes, and increased circulating IL-6 was observed [115]. Some but not all, of these responses were Kupffer cell dependent. Independent of the capsid or expression cassette, scAAV vectors induced dose-dependent innate responses by signaling through TLR9 [115]. Other studies, as the removal of CpG sequences from the AAV genome or, the incorporation of TLR9 inhibitory sequences derived from telomeric DNA sequences, resulted in the attenuation of the immune responses [112,116,117].

In addition to TLRs, AAV genome, contains elements that can activate cytosolic DNA sensors, such as the ITR hairpin structures, that can be recognized by soluble PRRs in the cytoplasm, such as cGAS, IFI16, and AIM2 [17]. These bind DNA in a sequence-independent way but in a length- and structure-dependent manner.

As mentioned in a previous section, cGAS binds double-stranded DNA (dsDNA) or DNA-RNA hybrids preferentially longer than 36 bp. Although it is a cytoplasmic sensor, it was shown to exist at endogenous levels in the nucleus [112]. It dimerizes upon binding to DNA and triggers a cascade, involving STING and TBK1, to induce the transcription of type I IFN genes (through IRF3) and antiviral cytokines, such as TNF-α and IL-6 [118]. A recent study showed that AAV vectors induce the expression of DNA sensors including cGAS and antiviral genes, such as TNF-α and IFN-γ [119]. In that study, AAV transduction was sixfold higher in cGAS–/– mouse embryonic fibroblasts than in WT fibroblasts.

IFI16 exists in both the cytosol and the nucleus and is activated by both ssDNA and dsDNA [120]. It is a member of the pyrin and HIN (hematopoietic IFN-inducible nuclear) domain-containing (PYHIN) family of proteins and the preferential length of its ligand DNA is 70 bp [121]. In the nucleus, upon binding to viral DNA, IFI16 can move to the cytosol to activate the inflammasome pathway. IFI16 may cooperate with the cGAS/STING pathway in some contexts. IFI16 also silences viral gene expression by facilitating the heterochromatinization of the viral genome in the nucleus [122]. IFI16 expression has been shown in CD34+ cells from human bone marrow and monocytoid lineage cells [123]. IFI16 and cGas have additionally been shown to be able to induce cell cycle arrest and apoptosis or pyroptosis in certain cell types [112]. IFI16 was also described to negatively regulate p53 and p21 influencing p53-mediated cell cycle arrest [112].

AIM2, as IFI16 is a PYHIN family member, preferentially binds to DNA stretches of approximately 80 bp in length [121]. It can bind dsDNA in the cytoplasm, forming a NLRP3 inflammasome (a multiprotein complex that triggers caspase-1) and leads to the production of mature IL-1β and IL-18. AIM2 is known to sense DNA damage in the nucleus and induces inflammasome activation, whether AIM2 can sense viral DNA in the nucleus is unknown. Further studies are required to elucidate whether AAV infection activates the AIM2 inflammasome pathway [17].

AAV transduction also originates responses to DAMPs for which hematopoietic cells are particularly sensitive. The AAV DNA hairpin with a free DNA end, as the ITRs, can be sensed by DDR proteins in the nucleus [112]. Through co-localization experiments, it has been shown that a variety of DDR proteins co-localize with nascent vector genomes upon nuclear entry as they undergo second-strand synthesis in discrete nuclear foci. These proteins include NBS1, phosphorylated NBS1 (p-S343-Nbs1), Mre11, Rad50, and Mdc1 [112,124]. The Mre11–Rad50–Nbs1 complex is responsible for recognizing dsDNA nicks near the 5′end of a double-strand break. Exposure of cells to DNA-damaging agents, which upregulate DDR proteins has been shown to increase recombinant AAV vector transduction in the absence of adenovirus co-infection [125,126]. The innate immune and DNA-damage response pathways are indistinguishably linked. Cellular DNA sensors function to prevent cellular replication in response to, not only, viral infection, but also to DNA damage. The cGAS/STING pathway gets activated as a response to genotoxic stress due to DNA damage, and the magnitude determines whether cells will repair, go into senescence, or undergo cell death [112].

#### 3.1.2. Restriction Factors Sensing AAV Vector RNA Products

During the late stage of AAV transduction, dsRNA is generated as an AAV genome-derived replication intermediate that stimulates intracellular dsRNA sensors, including RIG-I, MDA5, and LGP2, resulting in the activation of NF-κΒ and IRF signaling, consequently promoting type I IFN production [127]. Both NF-κΒ and IRF signaling pathways stimulate the expression of numerous downstream genes that induce an anti-viral state and activate anti-viral adaptive immune responses. Blocking dsRNA activation pathways, including MDA5 and MAVS, was shown to inhibit IFN-β expression from AAV-transduced cells and increase transgene expression [128].

The AAV ITR has inherent promoter activity. The presence of 5′- and 3′-ITRs in AAV genomes can result in the production of both sense and antisense RNAs, forming dsRNA intermediates [17]. These dsRNA molecules are then the subject of recognition by cytosolic RLRs. Cytosolic RLRs are expressed in almost all mammalian cell types. Additionally, RIG-I and MDA5 were observed to be upregulated in the primate retina after long-term AAV transduction [128]. RIG-I recognizes 5′-triphosphorylated blunt-ended short dsRNA or ssRNA. MDA5 preferentially recognizes long dsRNA. LGP2 can regulate RIG-I and MDA5 signaling via its ability to bind RNA [17]. MDA5 and RIG-I have a common signaling adaptor, MAVS, known to induce type I IFN production.

#### 3.1.3. Restriction Factors Sensing AAV Vector Proteins

The AAV capsids are potential PAMPs for TLR recognition, such as TLR2 and TLR4, that can detect viral liposaccharides and glycoproteins. TLR2 has been reported as the cell surface sensing venue for AAV capsids in human liver cells [129]. In addition to TLR2, AAV capsids activate the unfolded protein response (UPR). However, capsid variants exhibit different levels of activation, likely due to the differences in cellular entry [130]. UPR activation may contribute to further enhance TLR signaling and activation of NF-κΒ pathways [127].

#### 3.1.4. AAV Vector Intrinsic Restriction Factors

The efficient cell entry of AAV vectors is mediated by the particle binding to ubiquitously expressed surface receptors and co-receptors that are specific to the viral serotype. Restriction of permissiveness occurs at the post-entry level, where multiple barriers constrain vector transduction. Several steps limit AAV vector transduction: entrapment of virions inside the endosomal/lysosomal compartments, inefficient nuclear translocation and uncoating, ineffective single-stranded to double-stranded genome conversion, and the poor stabilization of newly formed viral dsDNA (as single or concatemeric circular episomes) [131]. In the wild-type AAV, cellular co-infection is required by helper viruses. The latter modify the cellular environment of target cells to render them permissive for viral replication. In AAV vector transduction, the contribution of those helper factors is missing. Efficient transduction is mostly limited to post-mitotic cells, in particular, cardiomyocytes, neurons, retinal cells, and skeletal muscle fibers, and requires the use of relatively high multiplicities of infection [131].

AAV host-intrinsic RFs are poorly understood. Most studies are performed in the context of AAV vector transduction under the conditions discussed above, i.e., in the absence of helper virus factors and using high MOIs. Here, we review host intrinsic restriction factors associated with AAV transduction. Most of those target the (1) AAV DNA genome, hampering its conversion to double-stranded form, or the (2) AAV capsid, directing it to proteasomal degradation. Although the pathways have been identified, only part of their intervenient molecules have been discovered.

Among the few host proteins reported to block double-stranded DNA conversion are FKBP52 (and the U2 snRNP spliceosome complex), PHD finger-like domain protein 5A (PHF5A)), and U2 snRNP-associated protein.

FKBP52, or FKBP prolyl isomerase, when phosphorylated binds specifically with the D-sequence within the inverted terminal repeat (ITR) of the AAV genome [132,133]. FKBP52 can be phosphorylated at both tyrosine and serine or threonine residues, and phosphorylated FKBP52 inhibits the viral second-strand DNA synthesis, leading to inefficient transgene expression. Zhao and colleagues showed that dephosphorylated FKBP52 can no longer bind to the D-sequence, thereby allowing viral second-strand DNA synthesis and efficient transgene expression [132,133,134,135,136]. This was observed in hematopoietic and liver primary cells, and in cell lines. However, previous studies, using FKBP52-knockout (FKBP52-KO) mice, documented that the transduction efficiency in hematopoietic stem/progenitor cells and hepatocytes was significantly less pronounced than in mice, in which FKBP52 is dephosphorylated at tyrosine residues [134,135,136]. The authors suggest that dephosphorylated FKBP52, through interaction with HSP90, mediates the cytoplasmic transport of viral proteins to the nucleus [132]. FKBP52 is a cellular chaperone protein, ~80% is localized in the nucleus and ~20% in the cytoplasm, where it co-localizes with microtubules and dynein, a retrograde motor protein [137,138].

PHF5A, a component of the U2 snRNP mRNA splicing factor, was found to block the expression from recombinant AAV vectors [139]. PHF5A was identified among several candidates in a siRNA library screen study. The disruption of PHF5A expression specifically enhanced AAV vector performance in a serotype and cell type-independent manner. U2 snRNP proteins recognize incoming AAV capsids, through the direct recognition of intact capsids, to mediate the cellular restriction at the step after second-strand synthesis. U2 snRNP spliceosome complex was identified as a novel host factor effectively restricting AAV vectors. The genetic disruption of U2 snRNP and associated proteins, as SF3B1 and U2AF1, resulted in the increased transduction of AAV vectors [139].

The proteasomal degradation of AAV vectors blocks efficient vector transduction. Indeed, proteasome inhibitors were extensively described as enhancers of AAV vector cell transduction in many cell types, both in vitro and in vivo [140]. AAV2 surface tyrosines are targets of the epidermal growth factor receptor protein tyrosine kinase. The phosphorylation of these tyrosines, and subsequent ubiquitination, reduces AAV2 transduction. This hypothesis was tested by mutating key tyrosine residues on the AAV2 surface. These mutations showed to dramatically increase the AAV2 transduction in vitro as well as in vivo [141,142]. Additional mutations of threonines and serines also showed further enhancement in the transduction efficiencies of AAV2 vectors [143]. Moreover, the mutations of capsid residue targets of phosphorylation are not limited to AAV2, and are extensive to other serotypes [144]. Post-translational modification of the AAV capsid, either during vector production or during viral entry, can dramatically affect transduction efficiencies. Protein kinases, phosphorylating AAV capsids, targeting to ubiquitin–proteasome degradation, can thus be viewed as intrinsic restriction factors to AAV vector transduction.

Similar to proteasome inhibition, it has been shown that suppressing SUMOylation significantly increases AAV2 transduction [145]. Via a genome-wide siRNA screen, the proteins of the small ubiquitin-like modifier (SUMO) pathway were identified in Chen et al. to be critical in AAV restriction. The authors identified several members of the SUMOylation pathway, as putative genes interfering with AAV gene transduction. Ubc9 (the E2 conjugating enzyme), Sae1, and Sae2 were identified as factors involved in restricting the AAV vectors. Sae1 and Sae2 are the enzymes responsible for activating E1. The knockdown of those genes increased AAV transduction. In a following study, it is shown that the capsid protein VP2 can become SUMOylated [146]. The SUMOylation could be mediated by E3 proteins, as Trim33, or PIAS1, E3 ligases identified in the siRNA screen as putative AAV2 restriction factors [145].

Cellular stress responses, as DDR can trigger SUMOylation activity. AAV vector genomes are inducers of DDR. Several viruses can suppress SUMOylation activity by targeting the key components of the SUMO pathway, and among these are AAV helper viruses [147]. The production of AAV vectors in helper-virus-free HEK 293 have shown particle SUMOylation; however, the prevention of capsid SUMOylation does not rescue the restriction of AAV2 gene transduction. Thus, AAV vectors can become SUMOylated during infection. The incubation of AAV vectors within HeLa and A549 cells induced an elevated SUMOylation activity, which became visible as early as 8 hours post-transduction, and the effect increased over time. This effect, however, was only visible at MOIs of 105 or higher [146]. The authors attribute the restrictive effect of SUMOylation and its activation on AAV transduction via two processes: (1) capsid SUMOylation and the (2) activation of cellular protein SUMOylation by AAV infection [146]. One candidate for the latter is DAXX, a known antiviral factor [148]. In DAXX knockout cells, a high increase in transduction is observed.

**Table 2:** Host restriction factors involved in innate sensing AAV.

Viral infection Phase	Sensor	Ligands	Adaptors	Effectors/Effect
Entry/ Uncoating	TLR2/4	Capsid proteins	MyD88	Inflammatory cytokines/Type I IFNs
	TLR9	CpG DNA dsDNA, DNA/RNA	MyD88	Inflammatory cytokines/Type I IFNs
	cGas	dsDNA	STING	Inflammatory cytokines/Type I IFNs
	IFI16	ssDNA, dsDNA	STING/ASC	Inflammatory cytokines/Type I IFNs Caspase-1
	AIM2	dsDNA, ssDNA, (DD)	ASC	Caspase-1
	EGF Receptor tyrosine kinase (RTK)	Capsid	d.a.	Proteasomal degradation
	SUMO proteins	VP2, (DD)	d.a.	SUMOylation
Nuclear entry/ dsDNA synthesis/Transcription	cGas	DAMP response	STING	IFNs
	NBS1, Mre11, Rad50, Mdc1	(DD)	STING	IFNs
	RIG-I/MDA5	dsRNA, ssRNA	MAVS	Inflammatory cytokines/Type I IFNs
	FKBP52	AAV genome	d.a.	Abortive double strand conversion
	PHF5A	AAV capsid	d.a.	Abortive double strand conversion

d.a., direct actuators; DD, DNA damage.

Mano and colleagues performed a genome-wide RNAi screening to identify AAV vector transduction host RFs [131]. This study identified a high number of cellular factors restricting AAV transduction, with many having functions related to cell cycle regulation and DNA recombination and repair. Five of those proteins were: SETD8 (a SET domain containing lysine methyltransferase), CASP8AP2 (a component of the apoptotic machinery), SOX15 (a developmental transcription factor), TROAP (a cell adhesion molecule reported to mediate blastocyst implantation), and NPAT (a cell cycle progression regulator encoded within the ATM gene). Those proteins, when silenced, in addition to improved transduction, directly induced cellular DNA damage and checkpoint activation. Thus, they might function indirectly by inducing stress cell responses known to improve AAV vector transduction.

Several groups have made efforts to identify AAV transduction RFs. Madigan et al. (2019) identified in a CRISPR screening the apical polarity determinant Crumbs 3 (Crb3) as a key RF; and demonstrated that CRISPR knockout (KO) of Crb3 renders cultured hepatocytes more permissive to AAV [149]. They further demonstrated that Crb3 enables the sequestration of essential glycan attachment factors, but not the AAV receptor (AAVR) from the cell surface. Ablation of Crb3 disrupts tight junction integrity and cell polarity, resulting in the mislocalization of glycans to the cell surface, allowing viral attachment and entry.

CRISPR screens have also been used to identify host factors improving vector transduction. Meisen et al. conducted two independent, genome-wide CRISPR screenings in the U2 OS cell line, identifying GPR108 and TM9SF2 as critical host factors facilitating transduction [150]. Their full functions were not yet been elucidated; neither their interactor host proteins nor their connection with the innate cellular responses.

## 4. Concluding Remarks and Future Directions

Nucleic acids carry essential genetic information in all living organisms. Therefore, it is natural that the innate immune system has evolved to recognize foreign nucleic acids as a form of defense. Indeed, nucleic acid structures are the most widely recognized PAMPs. Gene therapy, being based on the transfer of nucleic acids, is subjected to the surveillance and response of the human innate immune system. Gene therapy vectors, either from viral or non-viral vectors, are thus targets of host RFs. Viral vectors, in addition to DNA and RNA recognizing PRRs, may also present protein motifs targeted by PRRs. The activation of PRRs leads to the generation of IFNs and inflammatory response cytokines, upregulating the antiviral innate immune response, and restricting vector transduction. Additionally, intrinsic antiviral RFs also recognize viral nucleic acids and proteins and can act directly on the viral infection pathway abrogating vector transduction.

Here, it was reviewed and discussed the host RFs that can block lentiviral and adeno-associated vectors transduction. Although, human cells are well equipped with RF sensors, several aspects should be taken into consideration, and the existing data should be carefully analyzed. Indeed, LV and AAV vectors often provide efficient transduction rates. Several parameters influence vector immunogenicity and cell permissiveness being some host-dependent, while the others are vector-dependent. Measures can also be taken to minimize the induction of an innate immune response.

Some of the host-dependent factors influencing vector immunogenicity are, the pre-existing immunity (since adaptive and innate immune responses are intertwined), the delivery mode (in vivo vs. in vitro) administration route, the targeted tissue, the patient age, existence of concurrent infections, and genetic background, among others [151]. The cell and tissue targets of gene therapy will be of utmost importance in the context of gene therapy, since they are the primary cell sensors that the vector should encounter. While some cells constitutively express PRRs and intrinsic antiviral RFs, others only express them when stimulated by IFNs. Off-targeting cells of the innate immune system, in in vivo therapies, as those derived from myeloid progenitors (e.g., dendritic cells and macrophages), is a secondary mechanism that will indirectly restrict transduction efficiency. In addition to difficulties that may exist in the transduction of stems cells, the activation of the innate immune response can also impair their cell fate and function.

Within the vector-dependent factors are: (1) the particle serotype/species/pseudotype, (2) promoters used, (3) CpG content, (4) vector purity, and (5) vector dose [151,152]. Vector dose and purity are interconnected since the use of higher doses may increase DNA or other contaminants carry over from the producer cells or manufacturing bioprocess (eliciting unspecific immune responses) [32]. In the particular case of AAV vectors, the use of high doses has been related to the vector immunogenicity found in clinical trials [151]. Higher vector doses also elicit DNA damage responses that will induce further innate immunity responses.

Lentivirus infections are extensively studied. Adeno-associated viruses, being non-pathogenic, are mostly studied in the context of gene therapy. Thus, much more information and consolidated knowledge exists for lentivirus than for AAV. Notwithstanding, data from the wild-type viral infection cannot be directly translated to vector transduction in the context of gene therapy. In LV and AAV vector-based therapies, innate sensing could be exacerbated by the absence of viral accessory and helper proteins known to help the escape from antiviral restriction factors. Additionally, the high vector doses are significantly superior to those of a typical initial infection. In contrast, the viral cycle is incomplete, since LV and AAV vectors are replication incompetent; as such, the production of viral genomes and proteins prone to RF detection does not occur.

Several strategies can be used to lower innate immunity responses against AAV and LV vectors, for example, CpG depletion, the addition of TLR- and cGAS-inhibitory sequences, the expression of RIG-I-inhibitory proteins, the use of Cyclosporine H, and the use of immunosuppressants [17,153,154]. However, efforts to improve AAV and LV vector transduction toward the use of lower doses are still needed and will greatly improve their therapeutic efficacy in the clinic.

## Data Availability

Not applicable.
